# Decellularized diseased tissues: current state‐of‐the‐art and future directions

**DOI:** 10.1002/mco2.399

**Published:** 2023-11-20

**Authors:** Xiang Li, Jianyang Shan, Xin Chen, Haomin Cui, Gen Wen, Yaling Yu

**Affiliations:** ^1^ Department of Orthopedic Surgery Shanghai Sixth People's Hospital Affiliated to Shanghai Jiao Tong University School of Medicine Shanghai China; ^2^ College of Fisheries and Life Science Shanghai Ocean University Shanghai China; ^3^ Institute of Microsurgery on Extremities Shanghai Sixth People's Hospital Affiliated to Shanghai Jiao Tong University School of Medicine Shanghai China

**Keywords:** cancer, decellularized, disease modeling, ECM, fibrosis

## Abstract

Decellularized matrices derived from diseased tissues/organs have evolved in the most recent years, providing novel research perspectives for understanding disease occurrence and progression and providing accurate pseudo models for developing new disease treatments. Although decellularized matrix maintaining the native composition, ultrastructure, and biomechanical characteristics of extracellular matrix (ECM), alongside intact and perfusable vascular compartments, facilitates the construction of bioengineered organ explants in vitro and promotes angiogenesis and tissue/organ regeneration in vivo, the availability of healthy tissues and organs for the preparation of decellularized ECM materials is limited. In this paper, we review the research advancements in decellularized diseased matrices. Considering that current research focuses on the matrices derived from cancers and fibrotic organs (mainly fibrotic kidney, lungs, and liver), the pathological characterizations and the applications of these diseased matrices are mainly discussed. Additionally, a contrastive analysis between the decellularized diseased matrices and decellularized healthy matrices, along with the development in vitro 3D models, is discussed in this paper. And last, we have provided the challenges and future directions in this review. Deep and comprehensive research on decellularized diseased tissues and organs will promote in‐depth exploration of source materials in tissue engineering field, thus providing new ideas for clinical transformation.

## Graphical abstract

ECM from cancers (exhibited in the right panel) is different from healthy ECM (exhibited in theleft panel). Vast ECM proteins (e.g., collagens, fibronectin, HA, laminin) deposit in large quantities in the cancer ECM. The signaling pathways have been activated by the pathologic ECM deposition, related in the processes of cancergrowth, cancer metastasis and angiogenesis to facilitate cancer development.

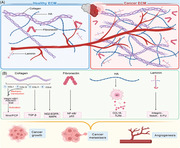

## 1 INTRODUCTION

With the improvement of medical level and the development of biotechnology, it is increasingly recognized that tissue engineering has played a cornerstone role in the development of regenerative medicine. Decellularized matrix, which is commonly used as a biomaterial for a variety of reconstructive surgical applications, has become a popular research topic in recent years.^1^ Maintaining of the native composition, ultrastructure, and biomechanical characteristics of extracellular matrix (ECM), alongside intact and perfusable vascular compartments, facilitates the repopulation of cells within the acellular organs. The major ECM proteins in various decellularized scaffolds, laminins, and collagens bind to β1‐integrin expression in repopulated cells, which enables site‐selective cell adhesion to physiological ECM domains.^2^ Despite perfect performances have not been established yet, the regenerated organ constructs have been demonstrated to exert certain physiological functions, thus providing a promising alternative treatment for patients suffering from organ failure. This technology has the potential to address the shortage of donor organs and the issues of organ rejection and immunosuppression associated with traditional organ transplantation.^3^, ^4^ Mammalian organism functioning as a native bioreactor impels the in vivo researches and applications of these ECM biomaterials. Implantation of these materials enrolls tissue‐resident and circulating stem cells, as well as other cell types to advance recellularization. This process also provokes HIF‐1α‐associated signaling pathways in the hypoxia microenvironment of the implanted areas, which facilitates angiogenesis and tissue/organ regeneration.^5^, ^6^ Furthermore, the immunomodulation mechanisms likewise function vitally during regeneration.^7^, ^8^ This has also been extensively reported in the urinary bladder matrix scaffold, one kind of decellularized ECM materials,‐mediated tissue defect repair after tumor resection.^9^ Although the decellularized materials facilitates proregenerative immune response, while in the context of tumor resection, several reviews have documented the applications of decellularized ECM‐based tissue repair in the context of tumor resection,^10^, ^11^, ^12^ highlighting the significance of decellularized biomaterials in the tissue engineering and regenerative medicine field.

Despite significant research progresses and promising application prospects, the development of decellularized biomaterials is limited to some extent due to the restricted harvest of native tissues and organs. Alternatives made from abandoned tissues/organs can serve as effective sources for decellularized biomaterials and can alleviate ethical concerns.^13^ It is widely known that deserted tissues and organs, especially those from human sources, have varying degrees of pathological changes. Notwithstanding evolutionarily conserved properties of ECM proteins, there are significant differences in the physiochemical properties of the ECM among tissues and organs in different physiological and pathological states. An overview of the main ECM components, including biglycan, hyaluronan, chondroitin sulfate (CS), and so on, that are responsible for ECM remodeling during several pathological conditions is provided by Karamanos.^14^ The development of bottom‐up proteomics approaches has identified the proteomic profiling of normal and pathological ECMs, including tumors and fibrosis.^15^ As illustrated in the review, the abnormal expression of specific ECM proteins (such as matrix metalloproteinases [MMPs] and a disintegrin and metalloproteinase with thrombospondinmotifs) is an important factor leading to the occurrence of pathological conditions. Unhealthy tissues/organs‐derived ECM may provide disordered physical integrity and atypical signaling molecules that can drive several biological functions.

Successful exploration and in‐depth understanding of the unhealthy tissues/organs‐derived decellularized ECMs will introduce technical innovations and provide new perspectives for biomaterial fabrication and application. Hitherto, current research on decellularized unhealthy tissues and organs has focused on cancer and fibrosis tissues (mainly fibrotic kidney, lungs, and liver). In this review, we provide deep and comprehensive review on decellularized diseased tissues and organs. The detailed preparation and pathological characterizations of decellularized diseased tissues/organs are discussed, and the innovative and advanced decellularization‐based models are emphasized, and last, the challenges and some future research directions are highlighted.

## 2 DECELLULARIZED CANCER MATRIX

Cancer, which occurs when abnormal cells grow uncontrollably in almost any tissue or organ of the body, remains the first leading cause of death.^16^ According to the latest data released by the World Health Organization, the number of global cancer diagnoses has reached 19.3 million, with the number of deaths increasing to 10 million in 2020.^17^ Breast cancer is the most commonly diagnosed cancer in the world, accounting for 11.7% of new cases, followed by lung, colorectal, and prostate cancer.^18^ The incidence rate of cancer in China provokes extensive attention. The National Cancer Center in China has reported that approximately 3.9 million people suffer from malignant tumors every year in China, with 2.3 million dying.^19^ Furthermore, China's cancer spectrum is currently in transition from the cancer spectrum of developing countries to that of developed countries. This means that lung, colorectal, and breast cancers are on the rise, while digestive tract cancers are on the decline. The researches on cancer arising therefrom, especially the detailed understanding of tumor microenvironment (TME), have an unprecedented significance. As a key component of TME, the ECM composing with complex and dynamic network of bioactive proteins can modulate the intercellular communications more than providing a physical and stable support to tissues.^20^, ^21^, ^22^, ^23^ During tumor development, the ECM undergoes significant alterations in compositions, structure, and dysfunctional biomechanical properties, which typically facilitate cellular transformation, angiogenesis, inflammation, invasion, and metastasis.^24^ In the following section, we have reviewed the characteristics of decellularized cancer matrix and their applications. The methods used for the decellularization of decellularized ECM from cancerous tissues will not be adequately illustrated here (Table 1). Briefly, ECM sources from cell or tissue sources are decellularized through physical methods (temperature and pressure), chemical methods (acid and bases), and enzymatic methods. These methods are suitable for tissues or organs of different densities. After reaching the decellularization standard, the obtained decellularized ECM substrate is then used for cell regeneration or whole organ perfusion to obtain 3D scaffolds with suitable volume and performance.^25^

**TABLE 1:** Decellularization methods applied in the decellularized cancer matrices.

	ECM sources	Decellularization method	Control sets	Experiment design	Results	References
Cell culture derived	MDA‐MB‐231	Freeze–thawing, 0.05% SDS, freeze–thawing	Healthy breast matrix scaffolds	MDA‐MB‐231 repopulation	Cell proliferation: MDA‐MB‐231‐ECM > healthy breast matrix scaffolds	^26^
				In vivo neogenesis	Tumor formation: healthy breast matrix scaffolds > MDA‐MB‐231‐ECM	
	MDA‐MB‐231, MCF‐7, MCF‐10A	0.5%Triton X‐100, 20 mM NH_4_OH, 100 μg/mL DNase I, 100 μg/mL RNase A	Matrices from MCF‐10A	Cells repopulation	Cell attachment: MDA‐MB‐231, MCF‐7 < MCF‐10A Chemoresistance: decreased on MDA‐MB‐231‐ECM	^27^
	HN12	Triton‐X/NH_4_OH	Matrigel, collagen I, fibronectin, laminin I	HN12 inoculation with cisplatin on matrices	Cell proliferation: carcinoma matrix > purified ECMs Chemoresistance: through Talin Regulation of NF‐κB	^28^
	NIH‐3T3, tumor‐associated murine fibroblasts	0.5% (v/v) Triton X‐100, 20 mM NH_4_OH	ECM from MCF‐10A, MCF‐7 and MDA‐MB‐231	Cancer cells inoculation on early and late ECMs	Cell proliferation: MCF‐10A(2D) > control 3D ECMs tumor‐associated/3D ECMs tumor‐associated matrices	^29^
	HT‐29, SW480, CCD‐841‐CoN	0.5% Triton X‐100 and 20 mM NH_4_OH	CCD‐841‐CoN‐ECM	Cancer cells inoculation	Chemoresistance: increased on HT‐29 cell‐derived matrices	^30^
	MDA‐MB‐231, MCF‐7, MCF‐10A, HT‐29, SW480, CCD‐841‐CoN inoculated substrates	0.5%Triton X‐100 and 20 mM NH_4_OH	Biocompatible polymer substrates	MDA‐MB‐231, MCF‐7, HT‐29, MCF‐10A, SW480, and CCD‐841‐CoN cells inoculation on corresponding inoculated substrates	Chemoresistance: tumor cell‐ECM‐treated substrates > untreated substrates	^31^
Animal derived	Pulmonary adenocarcinoma mice	Hypotonic Tris buffer, hypertonic Tris buffer, Trypsin, Triton X‐100, SDS	Matrigel group	MCF‐7, A549, SW‐480 and KYSE‐510 culture on the matrices	Cell repopulation rate: A549, SW‐480, MCF‐7 > KYSE‐510	^32^
	Metastatic liver and lung from breast cancer mice	Freeze–thawing, slice, Triton X‐100, 0.1%SDS	Healthy liver and lung	Implantation of ECM‐coated PCL scaffolds into bearing tumor mice	Tumor cell adhesion in vitro: metastatic ECM coatings > healthy matrix coatings Tumor cell colonization in vivo: metastatic ECM coatings > healthy matrix coatings	^33^
Patients derived	Brain tissues from GBM patients	0.1% Ammonium hydroxide and 1%Triton X‐100	Collagen‐based tumor model	GBM cells inoculation on GBM‐ECM hydrogels	Tumor cell invasion: GBM‐ECM tumor model > collagen‐based tumor model	^34^
	Rectal tissue from patient with rectal cancer	10 mM Tris, 0.1% EDTA, 50 U/mL DNase, 20 mM Tris, 2 mM MgCl_2_	Healthy matrices	Monocytes inoculation	Monocytes differentiation: tumor matrices: M1 in normal matrices, M2 in rectal cancer ECM Induction of colorectal cancer cell invasion: tumor matrices > normal matrices	^35^
	Human colon submucosa and metastatic liver	Colon submucosa:2%SDC, 1%Triton‐X100 Metastatic liver: freeze–thaw, 1% SDS, 1% Triton‐X100, 100 U DNase	Normal ECM	In vivo implantation of colonic cancer cells inoculated within ECM gels	Formation of tumor‐like vasculature: tumor ECM > normal ECM Cell proliferation: tumor ECM > normal ECM Glycolysis: tumor ECM > normal ECM	^36^
	Human breast cancer	Tissue slice, SLES solution	Monolayer MCF‐7 cells	MCF‐7 cells inoculation	Early cell apoptosis rate: recellularized scaffolds < monolayer MCF‐7 cells Expression of cancer stem cell‐related markers: recellularized scaffolds > monolayer MCF‐7 cells	^37^
	Human breast cancer	Tissue slice, 1% SLES solution	Normal breast tissue	ECM characterization and MCF‐7 cells inoculation	ECM biologic activity (MMP‐9, E‐cadherin): cancer ECM < normal ECM Vimentin, ZEB‐1 and Snail: cancer ECM > normal ECM EMT development: cancer ECM > normal ECM	^38^
	Tumor tissue from CRC patients	One to three detergent‐enzymatic treatment cycles	Normal tissue	HT‐29 cells inoculation	Invasion ability of HT‐29: CRC‐ECM > normal ECM IL‐8 expression: CRC‐ECM > normal ECM	^39^
	Gastric tumor	Tissue slice, DPBS solution and 1% SLES solution	Para cancer tissue	Nanoparticles (NPs) diffusion analysis	NP diffusion ability: high ECM density < low ECM density, soft ECM > stiff ECM NP diffusion coefficients: aligned structure > network structure	^40^

Abbreviations: PCP, planar cell polarity; MDA‐MB‐231, triple‐negative breast cancer (TNBC) cell line; MCF‐7, non‐TNBC cell line; MCF‐10A, the human normal breast cell line; NH12, oral squamous cell carcinoma (OSCC) cell line; NIH‐3T3, fibroblast cell line; HT‐29, highly malignant colorectal tumor cell; SW480, low malignant colorectal tumor cell; CCD‐841‐CoN, normal colorectal cell; A549, human pulmonary adenocarcinoma cell; KYSE‐510, human esophageal squamous cell carcinoma cell; CRC, colorectal cancer; GBM, glioblastoma; DNase, deoxyribonuclease; RNase, RNA enzyme; SDS, sodium dodecyl sulfate; SDC, sodium deoxycholate; EDTA, ethylenediamine tetraacetic acid; DPBS, Dulbecco's phosphate‐buffered saline; SLES, sodium lauryl ether sulfate; PCL, polycaprolactone.

### 2.1 Characteristics of decellularized cancer matrix

**FIGURE 1:**
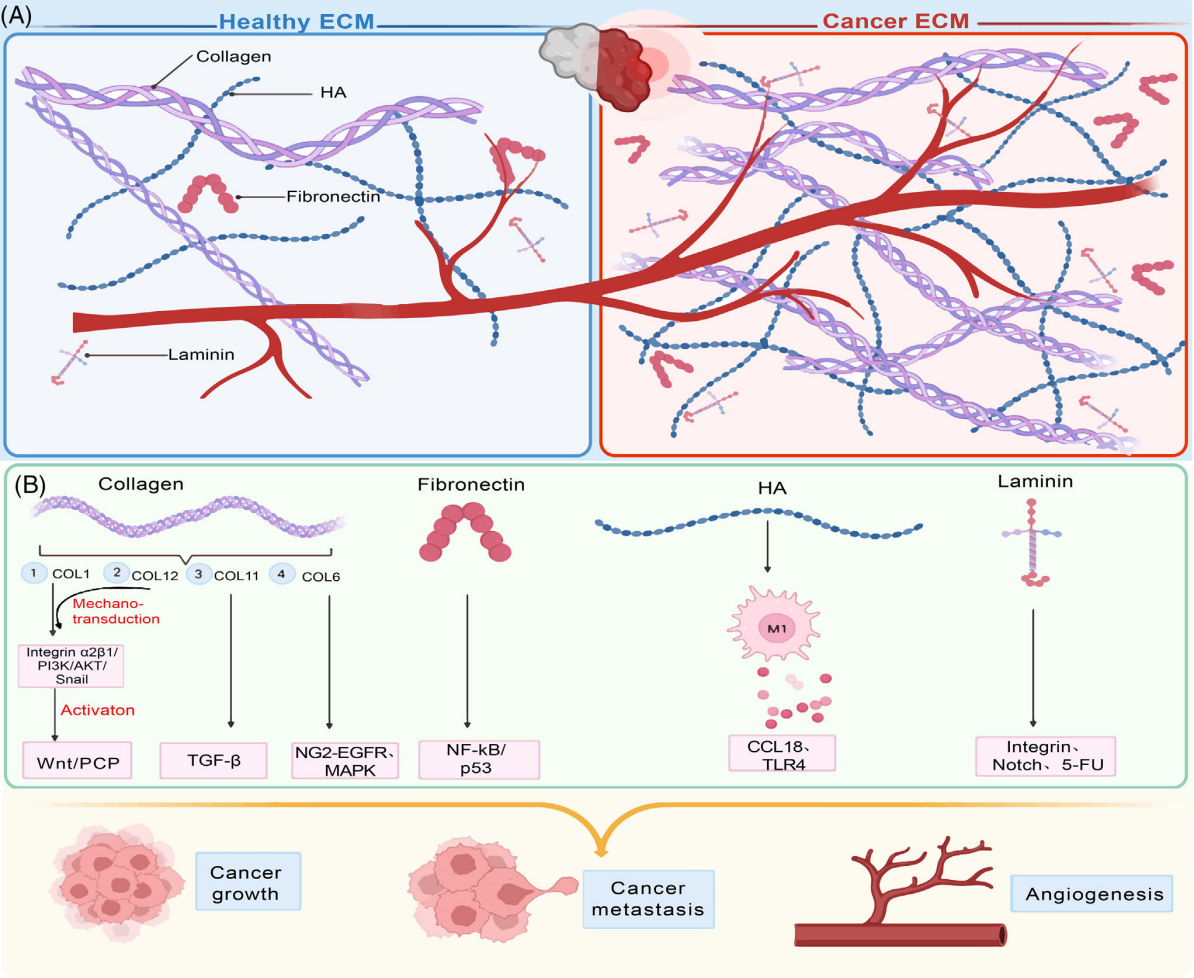
Compared with healthy extracellular matrix (ECM), ECM from cancers exhibits great differences. (A) The healthy ECM and cancer ECM are exhibited. In the left panel, vast ECM proteins (e.g., collagens, fibronectin, hyaluronic acid [HA], laminin, and so on) exist within the healthy ECM. In the right panel, almost all the ECM proteins deposit in large quantities in the cancer ECM. (B) The signaling pathways have been activated by the pathologic ECM deposition, related in the processes of cancer growth, cancer metastasis and angiogenesis to facilitate cancer development.

ECM is composed of more than 300 types of proteins and carbohydrates, including up to 19 types of collagens, proteoglycans (e.g., perlecan, aggrecan), over 200 types of noncollagenous glycoproteins (e.g., fibronectin, laminins, vitronectin, fibrillins), as well as growth factors and cytokines.^41^, ^42^ Romero‐López et al.^36^ conducted research comparing the healthy liver ECM to the metastatic liver cancer ECM of colorectal cancer (CRC), and significant differences were observed, particularly in ECM protein compositions, matrix stiffness, angiogenesis, and tumor growth. The characterization of decellularized intrahepatic cholangiocarcinoma (iCCA) exhibited that the heightened stiffness of tumor ECM is primarily caused by an upregulation of collagen fibers, though the levels of reticular fibers and elastic fibers within the iCCA ECMs are downregulated.^43^ While the proportions remain significantly different, the majority of ECM components derived from healthy tissues/organs are still found to be expressed within the cancer ECMs (Figure 1).^42^, ^44^

Collagen is an essential component to maintain the structural integrity of ECM. Studies have shown that Type I collagen (COL1) have a large amount of deposition in breast cancer, and it is precisely distributed in tumors.^45^ Excessive deposition of COL1 at the level of rectal cancer cell metastasis can activate integrin α2β1/PI3K/AKT/snail signal pathway^46^ and WNT/PCP pathway,^47^ thus promoting the metastasis process, and also promoting mesenchymal–epithelial transition (EMT), tumor growth, and increase the stemness of rectal cancer cells. Papanicolaou et al. have also revealed the key role of COL1 in TME, and has additionally demonstrated that COL12 secreted by cancer‐associated fibroblasts (CAFs) can establish a microenvironment that promotes tumor cell metastasis by regulating the mechanical transduction of COL1, which is closely related to the prognosis of tumors.^48^, ^49^, ^50^ This discovery indicates that COL12 may serve as an indicator of high‐risk cancer and may have a certain impact on the immunological progress of tumor. Moreover, the overexpression (OE) of A1 subtype of COL11 has also been proven to affect the phenotype of CRC via TGF‐β.^51^ Wishart and his colleagues^52^ conducted a study in which they decellularized breast cancer tissue and proved that obesity could change ECM components of breast tissue. This study also indicated that body mass index of patients and tumor matrix possess a complementary relationship.^52^ In addition, in this research, full‐length COL6 was also found to be upregulated in breast cancer tissue and associated with the migration of breast cancer cells through NG2–EGFR crosstalk and MAPK signal transduction pathways, proving that COL6 may be a new trigger for breast cancer specificity.

The vast protein modules contained within the tumor ECMs are concerned in tumor clinical characteristics, for example, tumor grade, which has been reported by Landberg et al.^53^ through high‐throughput sequencing methods. This study utilized patient‐derived scaffolds composed of 16 key proteins, including 12 core proteins associated with the secretion behavior of exocrine body, and the SCF–KIT signal pathway contained in module 1 may become a cancer treatment target, confirming that the binding of secreted proteins with decellularized scaffolds possesses the property of blocking the tumor promotion induced by TME. OE of laminins, such as laminin α1 and laminin α5, has been observed in tumors, particularly during CRC progression. These laminins can enroll CAFs to stimulate the expression of VEGFA via integrin α2β1–CXCR4 complex and upregulate Notch signaling pathway to facilitate cancer growth, angiogenesis, and metastatic spread.^54^, ^55^ Laminin γ2 is also involved in the proliferation, migration and invasion of CRC cells. While the lung adenocarcinoma metastasis process is attributed to the upregulation of laminin α2.^56^ Additionally, laminin α5 is confirmed to be associated in chemical resistance to 5‐fluorouracil (5‐FU) chemotherapy.^57^ Another noncollagenous glycoprotein, fibronectin, is also overexpressed within tumors and enhances the proliferation of cancer cells via activation of NF‐kB/p53 signaling.^58^ Its function goes beyond cell proliferation but tumor angiogenesis and metastasis. In terms of angiogenesis, tenascin‐C (TNC), which was also proved to induce integrin αvβ3‐mediated angiogenesis, is unique in promoting the development of CRC caused by colitis.^59^ As one kind of glycosaminoglycans (GAGs) presented highly in ECM, hyaluronic acid (HA) in the decellularized matrix of rectal cancer has been shown to polarize macrophages into anti‐inflammatory phenotype and promote cancer cell invasion through CCL18^35^ and TLR4.^60^ Moreover, it has been observed that fibrillins, Emilin, vitronectin, and endomucin are produced abundantly in CRC, while periostin, versican, thrombospondin‐2, and tenascin are found exclusively present in this type of cancer.^61^ Additionally, the composition of tumor ECM differs in tumors with different metastatic ability.^62^

### 2.2 Decellularized cancer matrix‐based applications

**FIGURE 2:**
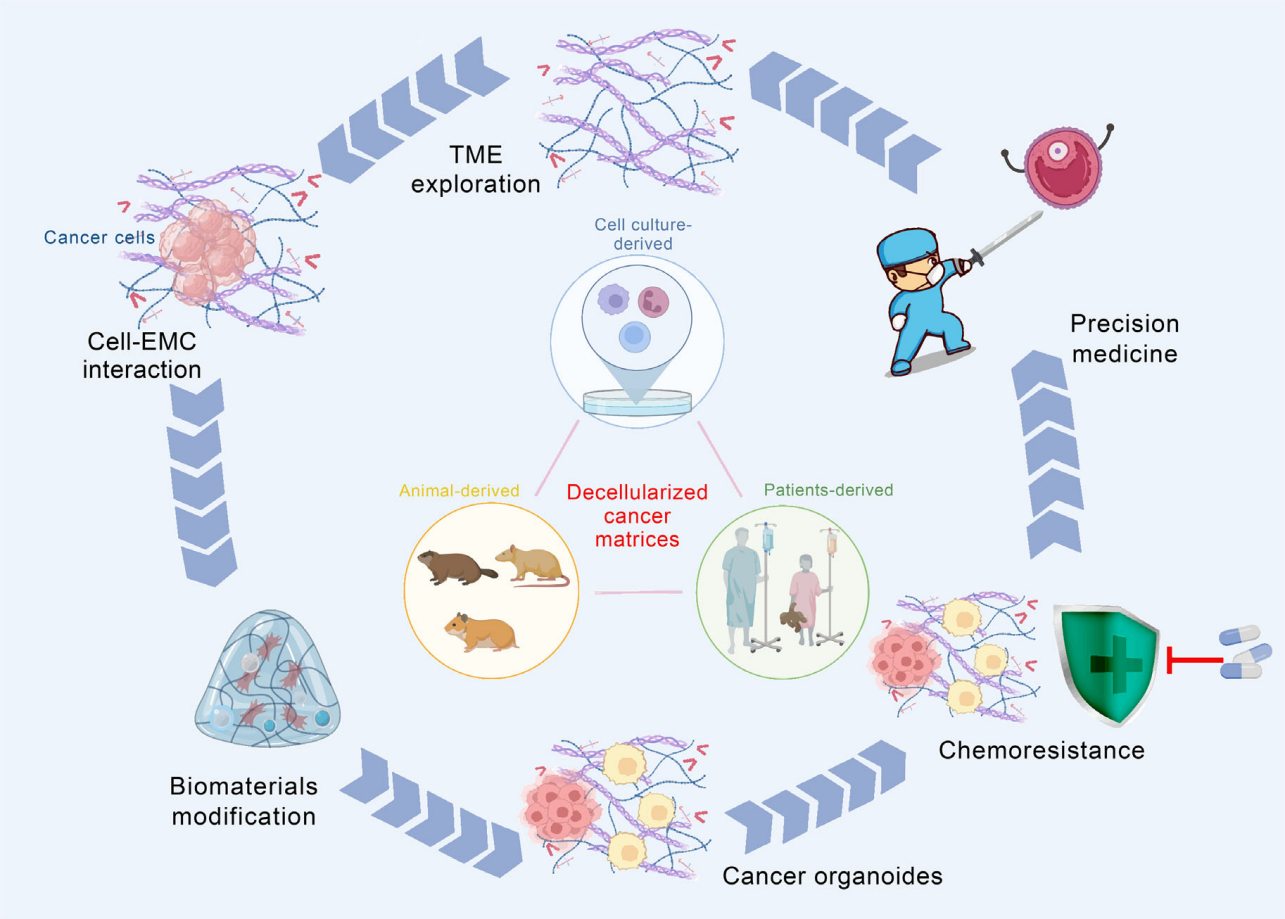
Decellularized cancer matrices are mainly derived from cancer cells, cancer tissues from animal models and patients. Deep exploration of decellularized cancer matrices can provide insights for TME exploration, interaction between cancer cells and ECM, modification of ECM‐based biomaterials, construction of cancer organoids, mechanism of chemoresistance, and precision medicine.

Communications between cancer cells and TME delineate cancer progression and therapeutic response (Figure 2). The rapid research progress of decellularized biomaterials has provided a solid foundation for the establishment of three‐dimensional (3D) in vitro tumor model, which has been confirmed effectively to investigate and illustrate how the mechanism blueprint of tumor ECM impacts cancer cell behavior and response to therapy.^63^ Compared with two‐dimensional (2D) tumor model, 3D tumor model has several advantages.^64^ It can highly imitate tumor matrix physicochemical properties, accurately regulate cell–ECM communication, and initiate cell invasion.^65^ Additionally, it preferably displays the structure of tumor and the complexity of TME.^66^ Decellularized tumor ECM‐based 3D tumor models have been shown to be a promising platform for exploring the growth of tumor cells in vitro, the release of supporting growth factors and the screening of related tumor genes.^38^, ^67^, ^68^ In the process of applying decellularized ECM to 3D tumor model, maintaining long‐term cell activity is crucial, and there are many factors affecting the results, such as different culture conditions (pH value, matrix mechanical properties, temperature, etc.) and organ species. Tissue‐specific microenvironment will improve tissue morphogenesis and affect long‐term cell viability in vitro. A growing number of studies have reported the importance of this condition.^69^, ^70^

Xiong et al.^71^ found that metastatic human breast cancer cells MDA‐MB‐231 and mice breast cancer cells 4T1 could invade and colonize in the decellularized lung matrix. Moreover, the native matrix‐mediated cancer cell invasion and colonization were demonstrated to be associated with the EMT process.^71^ Whereas nonmetastatic MCF‐7 cells were unable to survive in the native lung matrix, which is consistent with the findings of the research conducted by Jin and her colleagues.^38^ When repopulating cancer cells in human healthy breast ECM, they observed decreased cancer cell proliferation. However, under the communication with human breast cancer‐derived decellularized matrices, MCF‐7 cell increased EMT expression and cancer proliferation, which was obviously opposite from what was observed when the cells interacted with healthy ECM. It should be noted that the enhanced cancer cell proliferation caused by the decellularized cancer matrix is not a universal phenomenon. In another research, HT‐29 cells on 3D patients‐derived CRC scaffolds downregulated their ability of cellular proliferation.^72^ The decellularized patients‐derived CRC scaffolds remain abundant CRC associated ECM proteins. Their communications with human CRC cells HT‐29 increased the expression of pluripotency markers, which are closely associated with EMT and tumor mortality.^18^, ^72^ Peritoneal is the second most common site of metastasis in CRC after the liver,^73^ and peritoneal metastases (PM) is an important cause of the low survival rate of CRC.^74^, ^75^ Although ECM is important in regulating CRC metastasis homing to the peritoneum, the mechanisms underlying the interaction between metastatic cells and ECM remain poorly understood and a limited number of in vitro models are available for the study of the PM process. In recent reports, Varinelli and colleagues using an ex vivo 3D model obtained by combining patient‐derived decellularized ECM with organoids to mimic the metastatic niche showed that decellularized ECM in the peritoneal cavity allows the growth of organoids obtained from PM, facilitating the development of 3D nodules that maintain PM features in vivo. In this study, the organoids were preferentially grown on a scaffold obtained from the neoplastic peritoneal, characterized by a greater stiffness than the normal scaffold.^76^ This result may make an important contribution to the personalized treatment in the future.^77^ van Tienderen and his colleagues^78^ seeded patient‐derived cholangiocarcinoma (CCA) organoids (CCAOs) onto the decellularized CCA matrix (CCA‐M) to explore the detailed role of tumor ECM in CCA progression. They observed that the expression of stem cell markers, such as LGR5, CD44, and Wnt target gene CLDN2, was downregulated in the CCA‐M‐cultured CCAOs. On the other hand, the expression of AQP7P1, TCIM, ASIC4, and other transport proteins were upregulated alongside with the upregulation of ITGB2 antisense RNA1, ITGB8, and ITGB1, these genes expression suggested that the CCA ECM could drive the CCAOs toward desmoplastic matrix deposition and mesenchymal transition.^78^ Besides, the activation of CCA‐M‐mediated NF‐κB‐induced TNF‐α signal transduction pathways further consolidated the especial role and significance of decellularized cancer matrices in guiding tumor cell behaviors. Simultaneously, decellularized cancer matrices‐based cell researches provide a feasible alternative for tumor modeling, mechanism exploring and drug screening.^79^ Compared with decellularized matrices derived from healthy tissues and organs, the OE of COL3 α1 chain in the iCCA‐derived decellularized matrix enhanced the migration of tumor cells,^80^ indicating that the abnormally expressed ECM proteins within tumors are important factors in regulating the communication between tumor cells and decellularized matrices. In central nervous system tumors, the origin of tumors and changes in TME are closely related to the development and progress of diseases.^81^, ^82^ Glioblastoma (GBM) is a highly invasive tumor commonly found in the central nervous system. Koh and his colleagues^34^ have gained in‐depth understanding of the invasive characteristics of the GBM. They revealed that in the patient‐derived decellularized matrices‐based 3D model, inhibition of MMP 2/9 and HA synthase can change the morphology of GBM cells and its invasion mode. Inhibiting MMP2/9 and HAS both resulted in the inhibition of GBM cell proliferation and dissemination. Furthermore, when inhibiting MMP2/9, the dispersion of GBM cells is mainly rounded, whereas inhibiting HAS resulted in mainly elongated dispersion of GBM cells, this discovery of cell adaptability putting forward new insights into the expedition of drug resistance.^34^

Chemotherapy is often the first choice in the treatment of cancer, while the increasing chemical resistance poses significant challenges to the effective applications of chemotherapy.^83^ ECM remodeling has been literally effective to promote the acquisition of 5‐FU resistance from the perspective of EMT.^84^ During the interaction between cancer cells and decellularized tumor scaffolds, which are markedly distinct from those derived from healthy tissues, the administration of 5‐FU in the HT‐29 cells culture enhanced the expression of TGF‐β to induce EMT. And it furthermore increased the expression of ABCB1,^83^ one kind of ATP‐binding cassette transporters that have been demonstrated to be responsible for drug resistance. The cancer cell‐mediated 5‐FU resistance is not confined to a single cell type. Similarly, HT‐29 cells interacting with tumor ECMs could upregulate another marker associated in drug resistance, ABCB1.^83^ Mechanically, the tumor ECM‐facilitated 5‐FU resistance partly lies in the highly expressed CS chain, which has been demonstrated to promote the expression of ABCB1. An additional element dictating the chemotherapeutics resistance is the physical properties of tumor ECM. Among these properties, ECM stiffness has gone deeply into exploration.^85^ Lysine oxidase (LOX) has been proved to be the key enzyme of ECM regulation. For example, it has been found to stimulate tumor angiogenesis.^86^ In addition, it plays an important role in catalyzing the cross‐linking reaction of collagens and elastin, thereby modulating the ECM rigidity.^87^ Lv et al.^88^ reported that decellularized ECMs derived from MDA‐MB‐231 with different expression levels of LOX possess obviously different matrix stiffness while still retaining the main components of ECM. Different from the static stiffness of decellularized matrix reported previously, this study has established a dynamic stiffness gradient using LOX interference (IF) and OE lentivirus vectors to generate MDA‐MB‐231 cells with varying levels of LOX expression. The resulting solid tumors were decellularized to obtain 3D decellularized ECM scaffolds with a dynamic stiffness gradient. The study proved that the abundance of low‐stiffness decellularized ECM stent ECM was lower and the porosity was higher, which is conducive for the proliferation and migration of cancer cells. Furthermore, the study confirmed that the 3D decellularized ECM stent had good cell compatibility. Conversely, the decellularized scaffolds with high rigidity increase the drug resistance of MDA‐MB‐231 cells by upregulating the expressions of FAK, Bcl2, and especially the markers associated with drug resistance, including ABCB1.^88^ LOX protein‐like 1 (LOXL1) has been shown to inhibit the deterioration of rectal cancer by suppressing the activity of Yes‐associated protein,^89^ while LOXL2 has been found to promote the progress of CRC by blocking the cell cycle.^90^ Additionally, another protein cross‐linking enzyme transglutaminase‐2 thickens collagen fibers to facilitate the enhancement of matrix stiffness, thus negatively influence the prognosis of CRC.^91^

Other than cancer cells, nontumor cells also participate in the decellularized matrices‐based tumor modeling and drug resistance exploration. Tumor‐associated macrophages (TAMs), the most abundant innate immune population in TME, possess heterogeneity and differentiation plasticity.^92^ The differentiated antitumor TAMs preserve the characteristics of antigen‐presenting cells, including high expression of MHC‐II, phagocytic and tumor killing activities, and the production of proinflammatory cytokines that support and activate adaptive immune cells. On the contrary, protumor TAMs has immunosuppressive effects characterized by low expression of MHC‐II and high expression of inhibitory molecules such as PD‐1, PD‐L1, VISTA, B7‐H4, and Tim3.^93^ Coletta et al.^94^ inoculated macrophages onto the decellularized matrix of both healthy intestinal mucosa (HC) and mucosa derived from patients with CRC. The monocytes polarized by the CRC ECM exhibited a downregulation of proinflammatory gene CD86 and MHC‐II, while showing an upregulation of anti‐inflammatory/protumor gene CD206. Further, the scientists revealed that the targeted regulation of miR146b fragment and let‐7i miRNA on the histocompatibility complex class II trans‐activator was the main mechanism of the downregulation of MHC‐II, which further intercepted the activation of specific T cells to help CRC cells escape immunity. Nevertheless, this was in sharp contrast to the cells seeded on the HC‐derived ECM. As illustrated in researches that depict the immunomodulation of decellularized healthy matrices, polarization direction of macrophages induced by the healthy tissues‐derived ECMs hinges on the tissue specificity of the decellularized matrices.^95^ However, whether similar observations exist in decellularized cancer matrices‐mediated immunomodulation requires further investigation.

## 3 DECELLULARIZED FIBROSIS MATRIX

**FIGURE 3:**
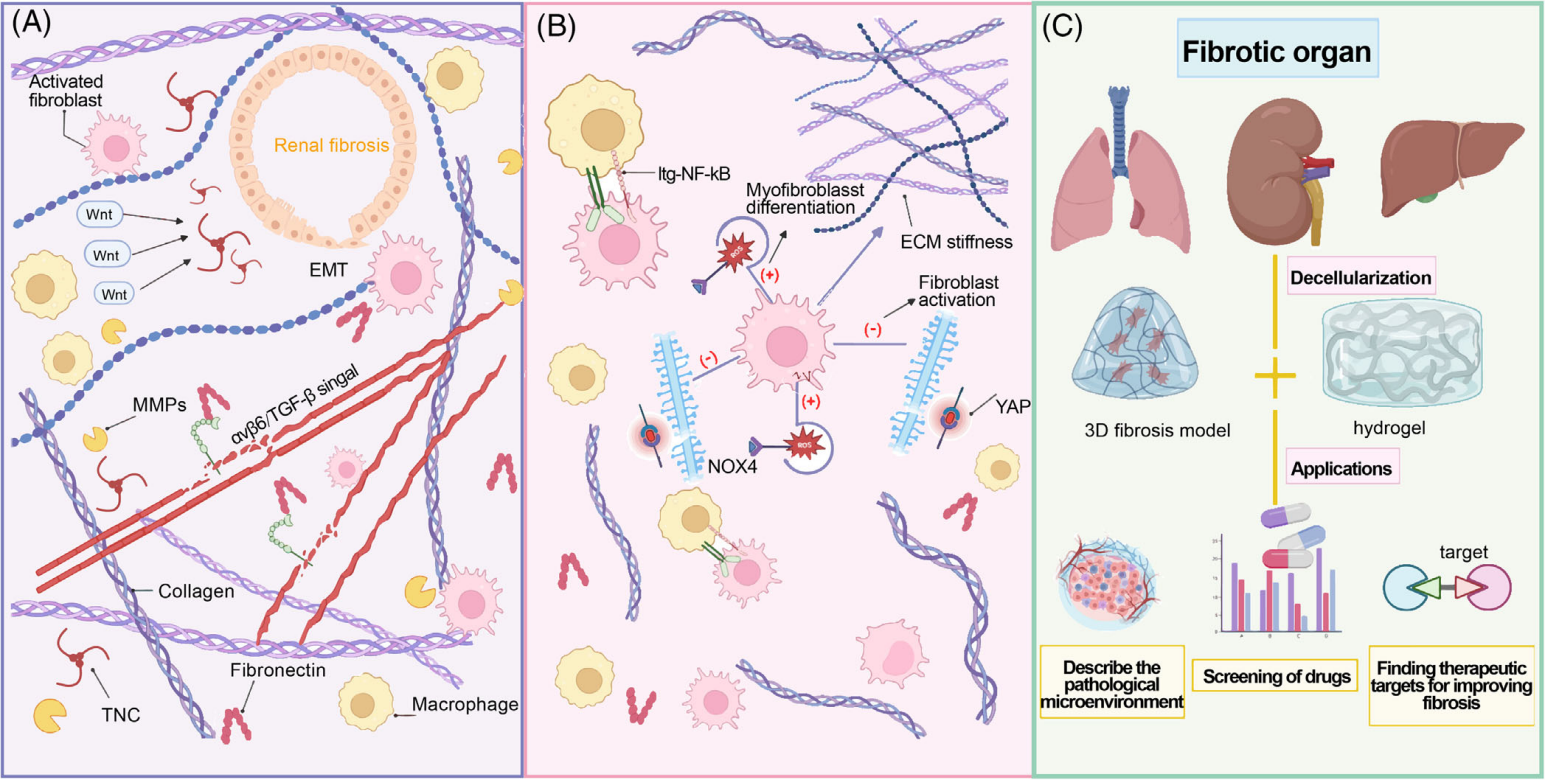
The characteristics and mechanisms of fibrotic ECM, as well as the application of decellularized fibrotic matrix. (A) The characteristics and mechanisms of fibrotic kidney ECM. In fibrotic kidney, collagen (type I and III) is significantly deposited, and tenascin‐C (TNC), which is almost undetectable in normal kidneys, exhibits enriched. TNC recruits tubular epithelial cells in fibrotic ECM through WNT signalings by activating integrin αVβ6. The fibrillin‐1 (FBN‐1) is activated by the integrin αVβ6/TGF‐β signal to induce endothelial damage and vascular sparsity. (B) The characteristics and mechanisms of fibrotic lung ECM. In addition to collagen deposition, the level of reactive oxygen species (ROS) in ECM is elevated. NADPH oxidase 4 (NOX4) responds to the overexpression of ROS and induces the differentiation of myofibroblasts. The decellularized fibrotic lung‐ECM can inhibit the expression of miR‐29 in fibroblasts and negatively regulate (YAP), thereby affecting the stiffness of the ECM. (C) Application of acellular fibrotic matrix. 3D fibrosis model and modified hydrogel prepared by acellular fibrotic matrices can be used to describe the pathological microenvironment of fibrosis, screening of drugs and exploration of therapeutic targets for treating fibrosis.

Besides decellularized cancer matrices, fibrotic tissue‐derived acellular matrix is another major research focus of decellularized diseased tissues. Fibrosis is a refractory chronic disease that can occur in various tissues and organs, characterized by the continuous and excessive deposition of ECM proteins and a decrease of parenchymal cells.^96^, ^97^, ^98^ Continuous fibrotic progression can lead to structural damage and functional decline, which may eventually lead to organ failure, seriously threatening human health and life. In the United States, it has been estimated that approximately 45% of deaths are attributed to fibrotic disorders, such as kidney fibrosis and liver fibrosis.^99^ In the last few years, the “coronavirus disease 2019” (COVID‐19) caused by “severe acute respiratory syndrome coronavirus‐2” swept across the globe.^100^ Pulmonary fibrosis is the main pathologic change in patients with severe COVID‐19 infections, and even in individuals with initially mild or moderate disease.^101^ Consequently, in‐depth exploration of the pathogenesis and treatment strategies of fibrotic diseases has important clinical significance. The proposal of mechanobiology links biology with mechanics, providing a broad platform for the research of fibrosis diseases.^102^, ^103^, ^104^ As the key element of this platform, decellularized ECM can fundamentally better simulate the stiffness and protein composition of ECM. Therefore, the research of decellularized fibrotic tissues has been gradually carried out in recent years (Figure 3).^105^, ^106^

### 3.1 Decellularized fibrotic kidney matrix

**FIGURE 4:**
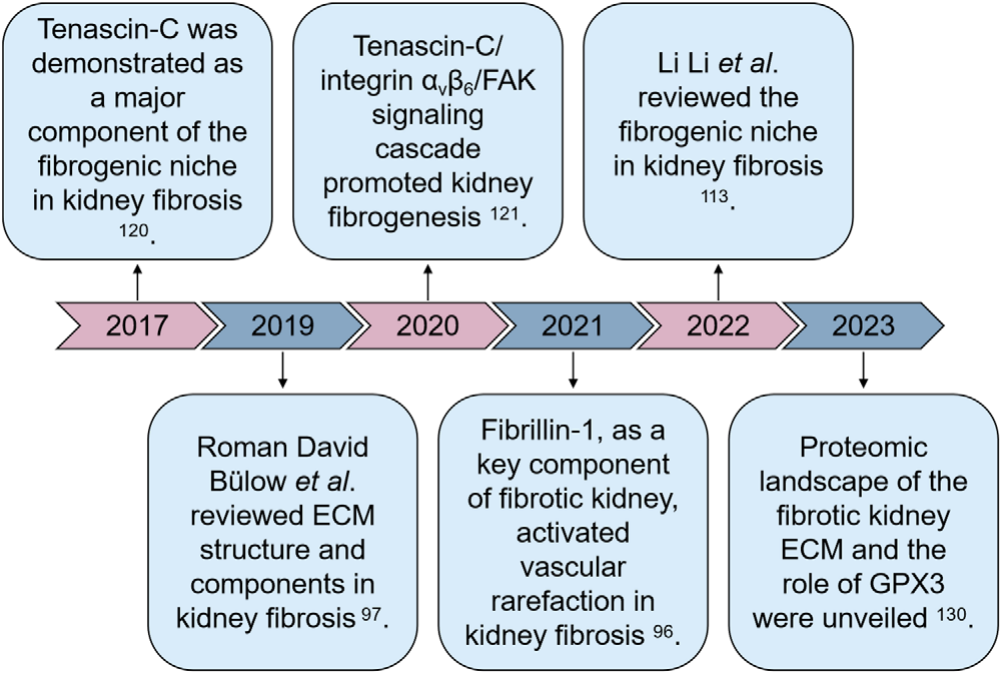
Brief history of decellularized fibrotic kidney studies. TNC as a major component of fibrotic kidney ECM was revealed in 2017. The structure and components of fibrotic kidney ECM were highlighted in 2019. Subsequently, other components and the relevant signaling cascade were unveiled.

Kidney fibrosis is the main hallmark manifestation of chronic kidney disease (CKD) that affects more than 10% of the world population,^97^, ^107^ and it is noteworthy noting that the distribution of renal fibrosis lesions is not uniform. Moreover, the safety, immaturity, and limited vascularization of kidney organoids are important issues. No existing protocol can produce kidney organoids that fully recapitulate the complex structure and function of the kidney, which limits the development of renal disease modeling and regenerative medicine.^108^, ^109^, ^110^ The application of decellularized kidney matrix is trying to improve the difficult problem of limited vascularization.^111^, ^112^ In fibrosis diseases, the concept of fibrosis niche has been proposed, which has been applied to a variety of organic fibrosis diseases,^113^ and the fibrosis niche contains enormous soluble factors effectively inducing fibroblasts activation, such as TGF‐β, fibroblast growth factor 2, interleukin 6, WNTs, sonic hedgehog gene, and so on, and embraces specialized ECM structures providing physical support.^114^, ^115^ In follow‐up study, it was found that the application of decellularized ECM scaffold derived from the fibrotic kidney proved that the fibrotic niche can promote the activation of macrophages, the proliferation of fibroblasts and the consumption of endothelial cells.^96^, ^116^, ^117^, ^118^ Obesity is an important factor in adipose tissue fibrosis.^119^ During the process of obesity formation, stromal vascular cells, preadipocytes, adipose‐derived stem cells surround large lipid‐laden mature adipocytes, allow the response of hypoxic conditions during obesity morphogenesis, and promote the development of fibrosis. In addition, many other factors, including genetic variations and environmental factors, may profoundly affect the pathological changes in the development of fibrosis. These cellular responses indicate that the decellularized fibrotic kidney matrix promotes and exacerbates the pathogenesis of CKD. Given its importance, it is urgent to clarify the composition of ECM network in the fibrotic niche. Li et al.^113^ compared fibrotic kidney tissues with healthy kidneys using decellularized kidney tissue scaffold (KTS) from fibrotic kidneys and healthy kidneys made by decellularized technology. Using an unbiased mass spectrometry proteomics approach, they identified approximately 1000 proteins in fibrotic tissues, which were divided into four major types: stromal cell protein, decellularized matrix protein, proteoglycan, and matrix modified protein.^113^ These proteins play an important part in the fibrogenic niche. Among others, stromal cell proteins have been proved to be the main proteins obviously upregulated in CKD–KTS, highlighting their importance in CKD progression. Wherein, TNC is highly expressed in renal fibrosis, while it is almost absent in normal renal tissue. TNC plays an important part in recruiting tubular epithelial cells WNTs and promoting the activation of fibroblasts and macrophages, thus facilitating the EMT process.^120^, ^121^ The application of decellularized fibrous scaffold conducted by Fu and his colleagues likewise demonstrates that TNC‐enriched microenvironment plays an important role during the transition from AKI to CKD via activating the integrin α_v_β_6_/FAK/ERK‐1/2 pathway, thereby triggering EMT and damaging the integrity of renal tubular cells. Emphatically, the increased TNC level in urine samples of CKD patients may serve as a noninvasive alternative drug to predict fibrosis.^121^ An analogous phenomenon is also observed in another type of stromal cell protein fibrillin‐1 (FBN1). In CKD, the FBN1 enriched in the ECM of fibrotic kidney can activate the integrin αvβ6/TGF‐β signal transduction pathway, leading to endothelial injury and vascular sparsity.^96^ The relief of endothelial injury and vascular sparsity after the suppression of FBN1 validates its pivotal role in the development of CKD. Other stromal cell proteins including TSP1, SPARC, SMOC2, and CTGF have been verified to regulate TGF‐β1 and also play irreplaceable roles in the formation of the fibrotic niche.^122^, ^123^, ^124^, ^125^, ^126^, ^127^, ^128^ Among ECM structural proteins of fibrosis tissue, COL1 and COL3 and fibronectin have been proved to deposit markedly in renal fibrosis tissue.^97^, ^113^ As a member of proteoglycan, polysaccharides play a key role in activating TGF pathway, and disaccharide interacting with TLR can promote inflammatory reaction that may aggravate the fibrotic course. In fibrotic kidney, the synthesis of ECM increases while the degradation decreases, which is related to the imbalance of MMPs. Relevant studies of CKD–KTS have reported that different types of MMPs play different roles in fibrosis. For example, MMP‐7 can serve as a urine marker and pathogenic medium of kidney fibrosis.^129^ In a newly published paper, Li et al.^130^ conducted a detailed investigation of CKD patients‐derived decellularized kidney scaffolds and revealed that glutathione peroxidase 3 (GPX3) was downregulated in the fibrotic kidney. Knockout of GPX3 could induce the expression of NADPH oxidase 2, promote the production of reactive oxygen species in the kidney, and activate p38 mitogen‐activated protein kinase. On the contrary, overexpression of exogenous GPX3 would alleviate renal fibrosis. This finding indicates the key role of oxidative stress in the fibrosis microenvironment, opening up a new way for the therapeutic strategies of treating the fibrotic kidneys. As one of the most common types of hereditary nephritis, Alport Syndrome often leads to kidney failure with extensive fibrosis. Scientists have reported the difference of ECM morphology and components between the decellularized kidneys from wild‐type mice and mice affected by Alport Syndrome. Compared with healthy kidney from wild‐type mice, decellularized Alport kidneys contain different fibrous protein deposition and cytokine content. Nevertheless, both healthy kidney scaffolds and Alport scaffolds activate macrophages toward prohealing (M2) phenotype. And the homogenization of the ECM from healthy and Alport kidneys decreases the anti‐inflammatory phenotype transition of macrophages, indicating that the 3D structure of fibrotic kidney ECM, rather than the component of the ECMs, is the key trigger of generating the anti‐inflammatory response^117^ (Figure 4).

### 3.2 Decellularized fibrotic lung matrix

**FIGURE 5:**
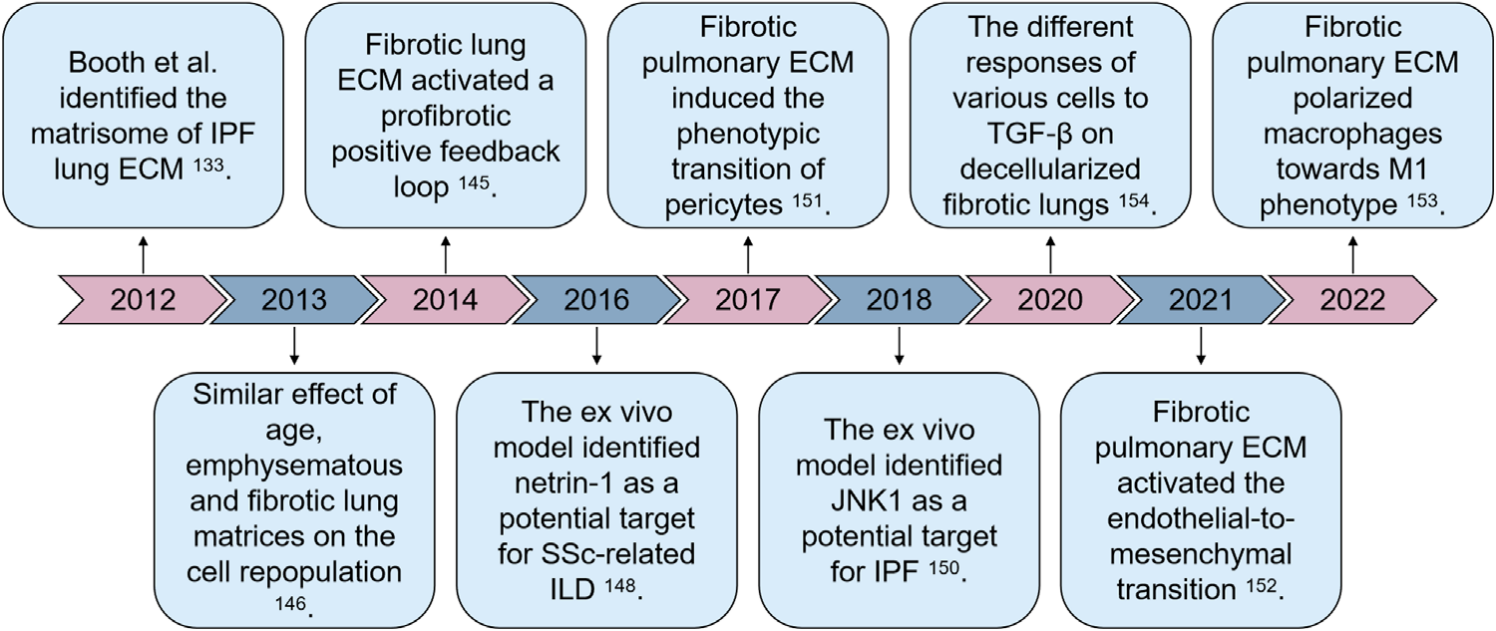
Brief history of decellularized fibrotic lungs studies. In 2012, the matrisome of fibrotic lung ECM from idiopathic pulmonary fibrosis (IPF) was first identified. Then, the cell responses to fibrotic lung matrices were studied in vast researches.

Idiopathic pulmonary fibrosis (IPF) is a pulmonary fibrosis model characterized by organ failure and gas exchange disorder, caused by irreversible destruction of lung structure.^131^ Currently, candidate drugs for the treatment of IPF are relatively limited,^132^ so it is necessary to establish an in vitro model using the decellularized lung matrix from patients (Figure 5). Booth et al.^133^ took the lead in conducting the matrisome analysis of lung ECM from IPF patients. COL3, COL6, GAG, and FBN1 obviously upregulated, and COL1, COL5, and COL15 only expressed within the acellular lungs derived from IPF patients. The alterations in ECM components are present in a region‐specific manner.^134^ Besides the different expressions of these structural ECM proteins, small‐molecular‐weight proteins in the diseased lung matrices, especially albumin, Itgb1, Apoa1, P4hb, and Fgg, are markedly elevated,^135^ potentially providing applications as biomarker candidates for lung fibrosis. Equally important, the heavy deposition of ECM components is bound to alter the physical characteristics of matrices. The matrix stiffness is given the main attention. As revealed by Booth et al.,^133^ the decellularized IPF lung scaffolds retained high matrix stiffness (approximately 16 kPa) compared with the normal lung ECM (about 2 kPa). In detail, the local stiffness within the fibrotic lung matrix is inhomogeneous.^136^ Similarly, generated ECM hydrogels from fibrotic human lung tissues reserves the high stiffness, which is equivalent to that of the native fibrotic lungs.^137^, ^138^ Attention has been paid to the important role of GAG in normal lung homeostasis and the pathogenesis of lung disease.^139^, ^140^, ^141^, ^142^, ^143^ However, knowledge about the functional role of individual GAG and their disaccharide composition in specific lung diseases remains limited.^139^ In addition, a recent comparison by Hoffman et al.^144^ of airway tissue from decellularized lungs obtained from COPD and IPF patients revealed a potential correlation between HS and CS disaccharide composition and their ability to bind and release FGF2 and TGF‐β, which is particularly altered in IPF lungs. These observations provide further information on understanding the functional role of ECM GAG in lung function and disease.^144^ Despite these achievement, more detailed exploration is needed to comprehensively dissect the physiochemical properties of decellularized fibrotic lung matrices, providing basis for fibrotic lung ECM‐based applications.

Fibroblast is the main executor of fibrosis. Its communication with abnormal lung matrices can depict the dynamic progression of disease, thus providing potential treatment targets for pulmonary fibrosis. The interaction of primary fibroblasts and aberrant lung ECM resulted in elevated gene expression of fibrosis.^98^, ^145^ The positive feedback loop remodeled the pathological progression of IPF. Importantly illustrated by the comparison of IPF‐derived primary fibroblasts and healthy lung‐derived primary fibroblasts, the origin of the ECM exhibited a more prominent impact on gene expression than did cell origin. Repopulated cells engrafted in alveolar spaces, while few cells penetrated into and adhered in the dense fibrotic regions of the bleomycin‐injured decellularized lungs.^146^ Cellular engraftment is attributable to focal adhesions.^147^ Despite great differences in the matrices among the lungs from old‐aged, emphysematous, and fibrotic mice, the behavior of cells repopulated did not differ between the different conditions. This is controversial with the data mentioned above, deserving further investigation. Additionally, increased apoptosis‐associated genes were observed with the culture period lasting, indicating the possible proapoptosis process of the decellularized fibrotic lungs. Compared with biochemical composition, the mechanical properties are also a more significant contributor to repopulated fibroblast differentiation.^138^

During the communications between fibroblasts and aberrant lung matrices, netrin‐1 has been identified to regulate cellular accumulation,^148^ indicating that it might be a novel therapeutic target in IPF. Besides, JNK1 is another potential target.^149^ In this connection, Kalafatis et al.^150^ made a step higher. They screened the proteins expressed by healthy fibroblasts repopulated onto the decellularized IPF. The elevated MMP7, MMP17, and PGF were associated with tissue remodeling, and the other proteins increased were related with inflammation and chemotaxis. Interestingly, the authors searched the expression of serum proteins in IPF patients. Protein–protein interaction analysis revealed that the elevated proteins in the ex vivo model and the serum proteins elevated in IPF patients were associated with disease severity and progression, providing additional evidence for potential systemic biomarkers for IPF. Theoretically, multiple cell types, other than fibroblasts, contribute to the initiation and progression of pulmonary fibrosis. While, hitherto, the ex vivo cell culture model based upon acellular fibrotic lungs has been reported minimally using other types of cells. Post the repopulation on the decellularized IPF lungs, human pericytes underwent a phenotypic transition, confirmed by the elevated expression of α‐SMA.^151^ While endotheliocytes behave differently. As it is well known that endothelial‐to‐mesenchymal transition is one of the main mechanisms of lung fibrosis. When engrafted on the decellularized ECM from bleomycin‐induced fibrotic lungs, the expression of α‐SMA in endotheliocytes was not observed.^152^ However, transcription factors SNAI1 and SNAI2/Slug were upregulated, indicating the activation of intermediate phage of the endothelial‐to‐mesenchymal transition. Additionally, macrophages, as one of the research highlights in the field of immunomodulation, have also been reported with the contact with fibrotic matrices.^153^ As it should be, all these explorations are to pave the way for pulmonary fibrosis treatment. The application of adipose tissue‐derived stromal cell conditioned medium (ASC‐CM) in the ex vivo culture model resulted in less ECM deposition.^154^ This research suggested that ASC‐CM probably inhibit fibrotic ECM‐induced profibrosis of fibroblast, and confirmed the efficiency of the ex vivo platform.

### 3.3 Decellularized fibrotic liver matrix

**FIGURE 6:**
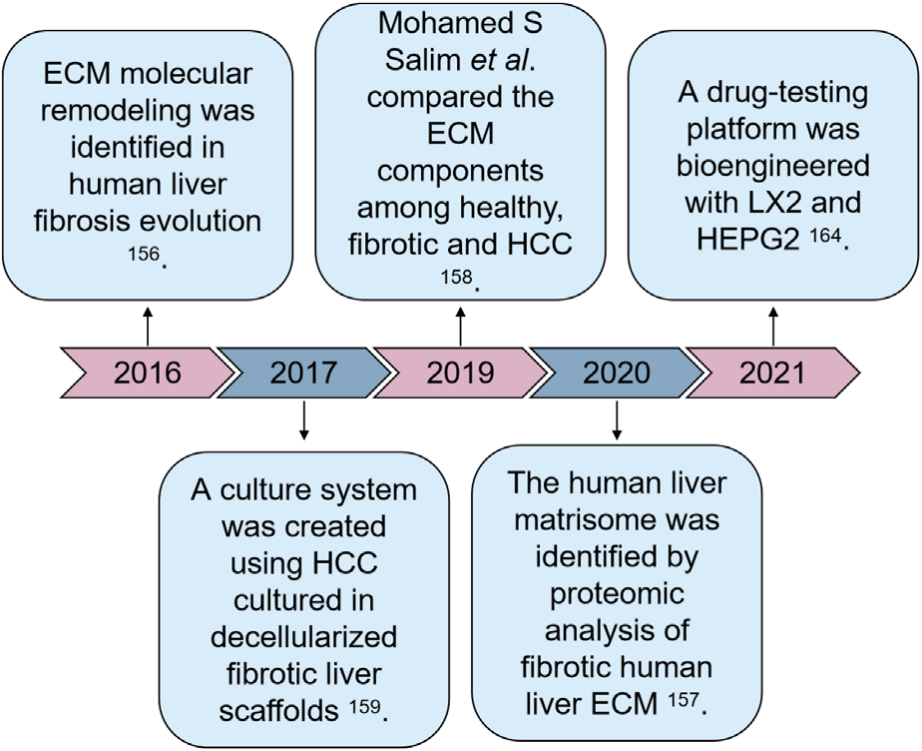
Brief history of decellularized fibrotic livers studies. Fibrotic liver ECM remodeling was first identified in 2016. Then, a culture system based upon decellularized fibrotic liver matrices was established, and a drug‐testing platform was bioengineered.

In parallel, liver fibrosis, which is the final common pathological course of chronic or iterative liver damage, has been studied deeply (Figure 6). In the research conducted by Naba et al.,^61^ the in‐depth proteomics analysis was employed to depict the atlas of human healthy liver ECMs. More than 150 type of distinct ECM proteins form the healthy human liver matrisome, including various collagens, 44 glycoproteins (e.g., fibrillins, fibronectins), 11 proteoglycans (e.g., biglycan, decorin), and matricellular proteins (e.g., periostin, tenascin‐C and ‐X). Great differences of ECM components lie between the fibrotic livers and healthy livers. The expression of COL1 in liver fibrosis is eightfold higher than in healthy livers, thus forming a dense matrix with the deposition of COL3, fibronectin, laminin, and so on in the Space of Disse.^155^ Baiocchini et al.^156^ classified the degree of liver fibrosis using the METAVIR system and characterized the ECM component changes of hepatic fibrosis through preparing decellularized liver scaffolds. They found that in the stage of moderate fibrosis, COL1 and COL3 increased slightly, along with increased COL12A1 and decreased COL10A1, COL14A1, COL16A1, and latent‐transforming growth factor beta‐binding proteins 1 (LTBP1). During the stage of severe fibrosis, COL1 and COL3 were also expressed in large amounts, while COL10A1, COL12A1, COL16A1, and LTBP1 showed the opposite expression pattern compared with those in the mild fibrosis. The characteristics of collagen spectrum in bridging fibrosis phase are slight transition from COL1, COL3, COL5 to COL6, as well as significant high expression of elastin, vitronectin, and microfibril‐associated glycoorotein 4 (MFAP4). However, the expressions of noncollagen components such as tenascin‐X and MFAP4 decrease during the transformation from severe fibrosis to bridging fibrosis. Cirrhosis, which is the lateral stage of fibrosis, is linked to poor survival and an increased risk of HCC. Daneshgar et al.^157^ identified 19 unique matrisome proteins (e.g., hemopexin, S100‐A8, inhibin beta C chain), which were completely exhausted in all stages of hepatic fibrosis, compared with healthy counterpart. Eleven matrisome proteins (e.g., LTBP2, Wnt‐7b, host cell factor 1, and hemicentin‐1) involved in the ECM remodeling were unique for only one of the different stages of liver fibrosis. It is famed that liver fibrosis is the initiating factor in the occurrence of hepatocellular carcinoma (HCC). Compared with decellularized murine HCC livers that exhibited significantly overexpressed collagen, fibronectin, and laminin deposition in both capsule and blood vessels, decellularized hepatic fibrosis samples showed higher thickness of laminin deposition in both capsule and blood vessels.^158^

Decellularized fibrotic livers have been employed for reproduction of the native physiopathological microenvironment of liver fibrogenesis and cancer, as well as for antifibrotic and even anti‐HCC drug screening. Repopulation of HCC cells on the decellularized fibrotic livers from CCl4‐treated rats for 1 week resulted in promoted cellular proliferation and an EMT phenotype, along with obviously increased chemoresistance.^159^ Similar EMT observation was reported in a new in vitro model based on decellularized cirrhotic human liver 3D scaffolds with HCC cells repopulation.^160^ The scientists identified 72 upregulated proteins in the decellularized cirrhotic scaffolds, including Col5A1, Col10A1, LOXL1, TGF‐β‐related proteins (e.g., TGFB1I1, LTBP1, LTBP4), and integrin‐related proteins (e.g., FLNA, FBN1, FBLN5). They confirmed that the high concentration of endogenous TGF‐β1 released from the cirrhotic scaffold facilitated Smad2/3 phosphorylation, highlighting a unique enhancement in EMT genes. In the fibrotic livers, great majority of hepatocytes are sensitive to TGF‐β1. TGF‐β1 induces and promotes the paracrine pathway of liver cells that is common in liver diseases through the mechanism of producing reactive oxygen species and initiating DNA damage response.^161^, ^162^ Furthermore, TGF‐β1 can inhibit the antifibrosis immunity of the liver by regulating the differentiation of regulatory T cells.^163^ Additionally, Thanapirom et al.^164^ proposed a novel 3D coculture platform with decellularized scaffold obtained from patients with liver cirrhosis. Coculture with LX2 and HEPG2 on the decellularized human cirrhotic livers increased profibrogenic markers and cancer phenotypic genes. The administrations of Sorafenib in the coculture platform demonstrated that the specific ECM‐dependent profibrogenic effect was associated with the promoted STAT3 phosphorylation. While the anticancer efficacy of Regorafenib was obviously decreased in the coculture system. Hitherto, the cell models based upon decellularized hepatic fibrosis is not enough yet to completely recapitulate the physio‐pathological microenvironment, and more works are needed.

In addition to the three organs mentioned above, the acellular fibrosis matrix of other tissues or organs is also being continuously explored. However, due to limitations in organ types and donor sources,^165^, ^166^ their research status is not as comprehensive as the three organs mentioned above. Heart attacks are a global health problem,^167^ resulting in significant morbidity, mortality, and healthcare burden. Adult hearts have a very limited capacity to regenerate after injury.^168^ Despite significant progress in early diagnosis and prevention, the fatality rate of heart failure has long been too high.^169^ Therefore, research of the pathological microenvironment of cardiac disease is necessary.^170^, ^171^, ^172^, ^173^, ^174^, ^175^, ^176^ For example, in the study of heart fibrosis disease, the feasibility of decellularization of the pathological heart tissue was demonstrated,^177^, ^178^ and by comparing the effects of decellularized natural ECMs from normal (dECM‐NH) or failing hearts (dECM‐PH) on human cardiac stromal primary cells. When seeded on dECM‐PH, CPC upregulated proremodeling cytokines (IGF‐2, PDGF‐AA, TGF‐β) and oxidative stress molecule H_2_O_2_. dECM‐PH is associated with impaired support for angiogenesis by paracrine secretion, as well as increased expression of the vascular endothelial growth factor (VEGF) chelating bait subtype of the KDR/VEGFR2 receptor. This result indicates that the resident CPC portion exposed to the pathological microenvironment that reshapes ECM loses its paracrine angiogenic properties and releases more profibrotic cytokines.^179^

## 4 CHALLENGES AND FUTURE DIRECTIONS

Despite the great advance in deep researches of decellularized ECM, mainly derived from healthy tissues and organs, the exploration of acellular diseased tissues and organs is still superficial, with lots of obstacles. At present, the research progress of decellularized diseased tissue still cannot be widely invested in clinical treatment.

### 4.1 Standardization of decellularization protocol

Biomaterials prepared from acellular matrix are currently widely used for animal‐derived medical devices and tissue engineering medical devices, such as artificial skin, biological valves, and acellular patches.^180^, ^181^, ^182^, ^183^ Similar to the decellularized healthy tissues and organs, decellularized biomaterials derived from diseased tissues/organs likewise face the challenge of standardizing the decellularization protocol, and this challenge is more prominent in ECM biomaterials derived from diseased tissues. Compared with the decellularized healthy tissues, subjects derived from diseased tissues/organs are confronted with the individual difference variable. When dealing with ECM from patients with cancers, it is essential to consider the different sources of tumor tissue donors, tumor grading, and the possibility of introducing adjustments in composition and structure. Therefore, it is necessary to characterize the properties of decellularized matrices in healthy, disease progression, and the final pathological state of tissues and organs to recapitulate the physio‐pathological microenvironment of cancers.^53^ Although the number of cancer tissues available for patient is limited,^24^ the development and applications of animal models has helped to address this issue. What is more important, a librarian based upon clinical cancer samples is indispensable alternative to carry out the researches. In addition, the dynamic nature of TME–ECM, the characteristics of disease‐specific mechanics and biomolecular complexity will establish an abnormal feedback loop.^184^ If not handled properly, it will lead to further imbalance of cancer cells and related tissues.^185^ In the preparation of decellularized matrices‐based hybrid biomaterials, cross‐linking reaction is essential, but overreaction has been proved to have adverse effects on the composition and biological activity of the matrix.^186^ Therefore, the selection of appropriate cross‐linking agent is a necessary issue to be considered when establishing 3D in vitro models. It should also be noted that the selection of cell types, the distribution of cells in the decellularized scaffold, and the repeatability of cell regeneration efficiency should be considered.^187^

Although the research of decellularized fibrosis matrix has been carried out in a large amount, there are still inevitable unpredictable challenges. For example, similar to the problems encountered in cancer matrices, there is a lack of sufficient donor sources for research of fibrotic tissue‐derived decellularized ECM.^188^ The size and feasibility of the produced decellularized scaffold is also one of the bottlenecks.^189^ In addition, the relevant knowledge about the interaction mechanism between the resident cells of fibrotic tissue and the surrounding environment is limited.^190^, ^191^, ^192^ In the fibrotic niche, the ECM network releases various growth factors and extracellular vesicles to form the fibrotic microenvironment, and the extent of the influence of the etiology of fibrotic diseases on the composition and dynamics of the fibrotic niche remains to be further determined. Furthermore, the determination of strategies to fight and even destroy the fibrotic niche, as well as effective treatment methods to prevent and treat fibrosis, are still unsolved problems.

### 4.2 Development of biomimetic scaffolds

With the rapid development of tissue engineering, it has become possible and necessary to prepare biomaterials with better performance. Although acellular matrix biomaterials have strong biocompatibility and excellent tissue regeneration induction performance, their mechanical and degradation properties may not fully meet the needs of tissue repair, and the needs of tissue repair caused by different disease types and tissue types also put forward different requirements for the various properties of acellular biomaterials.^193^, ^194^ Therefore, the modifications of biomaterials based on decellularized matrices, with ensuring their excellent biological performance, regulating their structural stability, mechanical properties, and antidegradation properties, and modifying their surface properties, pose new requirements for researchers.

Polymer brush is a promising grafting approach for the modification of biomaterials,^195^ with one end of polymer chain connecting to the active groups on the surface of materials in the form of covalent bond, and the other end extending in the direction perpendicular to the surface of the material to form a tightly ordered, brush like collection of polymer chains. In existing reports documenting the modification of the decellularized ECM materials, methacrylation is a widely used method.^196^, ^197^, ^198^ The methacrylic anhydride can react with the amino groups within the decellularized ECM, and then the application of UV irradiation can achieve the polymer surface modification. However, there is still lack of information for the methacrylation of decellularized diseased ECM materials. In addition, surface heparinization of acellular biomaterials has been a hot research direction in past years.^199^, ^200^ Post implanted in vivo, the surface heparinized biomaterials can activate local anticoagulation mechanisms and recruit various components in the circulatory system to actively participate in tissue repair. Besides, surface biotransformation, binding of chemically active groups, and surface modification are also optional methods for the modification of decellularized diseased ECM‐based biomaterials.

Vast research directions, including the standardization of decellularization protocols and the modification of acellular materials, guide the transformation of scientific researches into preclinical and clinical applications.

## 5 CONCLUSIONS

With the booming of experimental technologies, diseased tissues/organ‐based decellularized matrices have emerged in recent years, making up shortfalls against the monotony of tissue origin for the preparation of decellularized matrices. Recently, scientists removed cells from tissues and organs with diseased pathological conditions. In this review, we have reviewed the development of decellularized matrices derived from cancer and fibrosis. Contrastive analysis conducted between the decellularized diseased matrices and decellularized healthy matrices penetrates the characteristic of extracellular microenvironment of diseased tissues and organs. This provides a novel research perspective for revealing the occurrence and progression of diseases. Furthermore, constructing in vitro 3D cell culture models based upon decellularized diseased matrices recapitulates the mutual communications between types of cells and pathological ECMs and offers accurate pseudo models to exploit new strategies for cancer and fibrosis treatments. Despite great advances achieved, vast challenges mentioned above are still present. However, with the optimization of decellularized and tissue engineering technology, the application of decellularized diseased matrices with good biocompatibility and reliability can lead to more accurate simulation of physio‐pathological syndromes, thus expanding the potential applications of decellularized materials in medical settings.

## AUTHOR CONTRIBUTIONS

Xiang Li written and edited the original manuscript. Jianyang Shan edited the manuscript and drawing all the figures. Xin Chen edited the manuscript. Haomin Cui provided figure modification, manuscript modification, and funding support. Gen Wen provided the investigation and funding acquisition. Yaling Yu provided project administration, funding acquisition, and edited the manuscript. All authors have read and approved the final manuscript.

## CONFLICT OF INTEREST STATEMENT

The authors declare that they have no competing financial interests or personal relationships that could have influenced the work that was reported in this paper.

## ETHICS STATEMENT

Not applicable.

## Data Availability

Not applicable.
